# Management of anti-seizure medications in lactating women with epilepsy

**DOI:** 10.3389/fneur.2022.1005890

**Published:** 2022-11-17

**Authors:** Rong Yan, Jinmei Tuo, Zhenzhen Tai, Haiqing Zhang, Juan Yang, Changyin Yu, Zucai Xu

**Affiliations:** ^1^Department of Neurology, Affiliated Hospital of Zunyi Medical University, Zunyi, China; ^2^Department of Nursing, Affiliated Hospital of Zunyi Medical University, Zunyi, China; ^3^The Collaborative Innovation Center of Tissue Damage Repair and Regeneration Medicine of Zunyi Medical University, Zunyi, China

**Keywords:** epilepsy, women, breastfeeding, anti-seizure medications, adverse reactions

## Abstract

Epilepsy is a common neurological disease. At present, there are about 70 million epilepsy patients in the world, half of them are women, and 30–40% of women with epilepsy are of childbearing potential. Patients with epilepsy who are of childbearing potential face more challenges, such as seizures caused by hormonal fluctuations and the risk of adverse effects on the mother and baby from taking anti-seizure medications (ASMs). Breast milk is one of the best gifts that a mother can give her baby, and breastfeeding can bring more benefits to the baby. Compared with healthy people, people with epilepsy have more concerns about breastfeeding because they are worried that ASMs in their milk will affect the growth and development of the baby, and they are always faced with the dilemma of whether to breastfeed after childbirth. Regarding, whether women with epilepsy can breastfeed while taking ASMs, and whether breastfeeding will adversely affect the baby is still an important topic of concern for patients and doctors. This article reviews the existing research on breastfeeding-related issues in women with epilepsy to guide clinical practice, and improve the breastfeeding compliance of women with epilepsy.

## Introduction

As a common nervous system disease, epilepsy is characterized by repeated abnormal discharge of brain neurons (1). About 70 million people worldwide have been diagnosed with epilepsy. China accounts for one in seven, and half of epilepsy patients are women (2–4). In India, about 52% of 2.73 million epileptics are of childbearing potential, and about 500,000 women with epilepsy are of childbearing age in the US population (5, 6). According to the statistics, about 30–40% of women with epilepsy in the world are of childbearing potential (7). Compared with male patients with epilepsy, women face more challenges, such as pregnancy, seizures due to hormone fluctuations, fertility, lactation and menopause (8). Women with epilepsy pay more attention to whether taking ASMs during childbearing age will lead to fetal development disorder, and whether breastfeeding can be allowed after childbirth, and whether taking ASMs during lactation will lead to impairment of infant function. Many epileptic mothers are worried that ASMs in their milk will affect the growth and development of their infants, and they always fall into the dilemma of whether to breastfeed after delivery. At present, the focus of attention of every epileptic mother and clinician is whether breastfeeding is feasible for women epileptic patients and whether breastfeeding will cause adverse effects on infants. Therefore, this article briefly introduces the issues related to the use of ASMs in women epileptic patients during lactation to guide clinical practice and promote clinical decision-making.

## Benefits of human breast milk

Breast milk is considered the main source of nutrition for babies before half a year old and is rich in a variety of essential immune components with anti-infective activities that play key roles in immune formation (9). The study recommends that infants are exclusively breastfed for the first 6 months after birth, followed by complementary foods and combined breastfeeding after half a year (10). High-quality breastfeeding has positive impacts on the health of infants and mothers. It can reduce the risk of infants suffering from severe lower respiratory tract infections, non-specific gastroenteritis, obesity, diabetes, leukemia, atopic dermatitis, asthma, and other diseases. At the same time, it promotes postpartum uterine repair in mothers and reduces the risk of breast cancer, type 2 diabetes, ovarian cancer and postpartum depression (9). Although breastfeeding has many benefits, it is less likely to breast-feeding because of the concern that ASMs excreted into breast milk may be harmful to infants (11, 12). The most common reason for women with epilepsy to stop breastfeeding is that they are worried about the damage to baby caused by ASMs in their milk. The lack of clear evidence and guidance on breastfeeding is a source of anxiety for women and healthcare providers (13).

## Metabolic pathway of ASMs and the influence of ASMs in human milk on infants

The distribution of ASMs *in vivo* is affected by drug plasma protein binding and drug lipophilicity (14). Most of ASMs are extensively metabolized through CYP450 and uridine glucuronide-glucuronyltransferase (UGT) pathways, while some drugs are directly excreted from the body without metabolism (gabapentin, pregabalin, etc.) (15). No matter whether drugs are metabolized or not, drugs may be excreted into human milk. Studies have found that the extent of ASMs entering breastmilk is affected by many factors, such as chemical properties of drugs, maternal serum drug concentration and maternal metabolic capacity (16). There are some methods that can calculate the situation that infants are exposed to drugs through breastmilk during lactation, such as breastmilk/plasma (M/P ratio) and relative infant dose (RID). M/P ratio represents the penetration degree of drugs from serum to breastmilk. If the value of M/P ratio is >1, it indicates that the drug level in breast milk is high (17). RID is to compare the drug dose that the infant is exposed to through breast milk with the drug dose of the mother (mg/kg/d). RID is a function of MP ratio and maternal drug clearance, and the safety threshold is 5–10%. However, RID higher than 10% is not necessarily a taboo for breastfeeding (18, 19). It should be noted that M/P ratio is not necessarily proportional to RID. For example, morphine M/P ratio is 2, while RID is only 3–8%. However, the phenobarbital MP ratio is <1, and the RID is >50% (18). A high M/P ratio does not imply that a large amount of a certain drug will pass into breast milk. We need to combine M/P ratio, RID, molecular weight, pharmacokinetics and other factors to analyze. In order to further assess the lactation risk of ASMs, Hale divided the drugs into five grades (L1–L5) according to their physicochemical properties, metabolic capacity and other factors (20). The specific meanings are as follows. L1: This is a drug that has been taken by a large number of breastfeeding mothers without any observed increase in adverse effects in the infant. L2: Drug that has been studied in a limited number of breastfeeding women and does not increase adverse effects in infants; L3: No controlled studies in breastfeeding women; risk of adverse effects in breastfed infants is possible, Or controlled studies showing only minimal non-threatening side effects; L4: There is positive evidence that there are risks to breastfeeding the infant or breast milk production, but despite the risks to the infant, the benefits of use in breastfeeding mothers may be acceptable. L5: There is significant and documented risk to the infant based on human experience, or it is a medication that has a high risk of causing significant damage to an infant. The drug is contraindicated in women who are breastfeeding an infant (20). These classifications can be understood as drug safety assessment. Most of ASMs belong to L2, L3, and L4 (20). However, some new ASMs, such as Rufinamide, Stiripentol, Perampanel, Brivaracetam, Cannabidiol, Eslicarbazepine acetate were not classified in the book due to the lack of relevant research. In this article, we have referred to the Lactmed database, Medications and Mothers Milk 2019 and other literature to summarize the metabolic pathway, pharmacokinetics of common ASMs and the impact of ASMs in breastmilk on infants (Table 1).

**Table 1:** Pharmacodynamics and M/P ratio, RID of some ASMs.

**Drug**	**Classification**	**T ½ (h)**	**MW**	**T–max (h)**	**Oral (%)**	**Vd (L/kg)**	**PB (%)**	**M/P ratio**	**RID (%)**
PHT	L2	22–40	252	4–12	95	0.5–0.8	89	0.18–0.45	0.6–7.7
FOS	L2	0.367–0.683(Im) 0.133–0.25 (Ia)	406	0.5	No	0.06–0.15	99	No	No
CBZ	L2	15	236	4–5	100	0.8–1.8	74	0.69	3.8–5.9
LTG	L2	29	256	3	98	0.9–1.3	55	0.562	9.2–18.27
LEV	L2	6–8	170	1	100	0.7	<10	1	3.4–7.8
GBP	L2	5–7	171	1–3	50–60	0.8	<3	0.7–1.3	6.6
OXC	L3	2	252	4.5	40	0.7	40	0.5	1.5–1.7
TPM	L3	21	339.4	1.5–4	80	0.7	15–41	0.86	24.68–55.65
PGB	L3	6	159	1.5	90	0.5	0	0.34–0.76	7.18
LCM	L3	13	250	1–4	100	0.6	15	0.83	No
CLB	L4	36–42	301	0.5–4	87	1.43	80–90	0.13–0.36	4.38–5.33
CLZ	L4	18–50	316	1–4	100	1.5–4.4	50–86	0.33	2.8141
ESM	L4	30–60	141	4	100	0.72	0	0.94	31.4–73.5
ZNS	L4	63	212	2–6	No	1.45	40	0.93	28.88–44.1
PRM	L4	5–18	218	1–2	90	0.5–1	25	0.72	8.4–8.6
VPA	L4	14	144	1–4	100	0.1–0.4	94	0.42	0.99–5.6
PB	L4	53–140	232	53–140	80	0.5–0.6	80	0.4–0.6	24
RFM (21)	No	8–12	238	5–6	≥85	0.7–1.1	30	No	No
STP (21)	No	4.5–13	234.29	1–2	≥90	Variable	99	No	No
PER (21)	No	70–110	349.38	0.5–1.5	100	77	96	0.13	No
BRV (22, 23)	No	7–8	212.29	2	100	0.5	17.5	0.71	No
CBD (24)	No	60	314	0.5–6	6	^*^	>94	No	No
ESL (21)	No	20–40	296.32	1–4	≥80	2.7	35	No	No

PHT, Phenytoin; VPA, Valproate; CBZ, Carbamazepine; GBP, Gabapentin; LEV, Levetiracetam; OXC, Oxcarbazepine; TPM, Topiramate; LTG, Lamotrigine; PGB, Pregabalin; PB, Phenobarbital; PRM, Primidone; ZNS, Zonisamide; ESM, Ethosuximide; LCM, Lacosamide; PER, Perampanel; BRV, Brivaracetam; ESL, Eslicarbazepine acetate; CBD, Cannabidiol; CLB, Clobazam; RFM, Rufinamide; CLZ, Clonazepam; STP, Stiripentol; FOS, Fosphenytoin; T1/2, Half-life; MW, Molecular weight of the drug; Vd, Distribution volume; T-max, The time from administration to the time when the drug concentration in the mother's plasma reaches the maximum water; PB, The percentage of maternal protein binding. If a drug is highly bound to protein, it cannot easily enter the milk compartment. The higher the binding percentage, the less likely the drug will enter the breastmilk. Oral, Oral bioavailability refers to the ability of drugs to reach the systemic circulation after oral administration; M/P ratio, Refers to the ratio of drug concentration in milk to plasma drug concentration, revealing the degree of drug aggregation in milk; RID, refers to the ratio of the dose of drug ingested by the infant through breast milk to the dose of the drug in the mother's body, which is a better indicator of the relative dose of drug transferred to the infant; No, There is no relevant data reported in the literature; Im, Intramuscular injection; Ia, Intravenous administration;

* A dose of 1,500 mg given take in the fasting state, the apparent distribution volume is about 21,000 L; Approximately 43,000 L after intake of a 6000mg dose; L2, Drug that has been studied in a limited number of breastfeeding women and does not increase adverse effects in infants; L3, No controlled studies in breastfeeding women; risk of adverse effects in breastfed infants is possible, Or controlled studies showing only minimal non-threatening side effects; L4, There is positive evidence that there are risks to breastfeeding the infant or breast milk production, but despite the risks to the infant, the benefits of use in breastfeeding mothers may be acceptable.

### Type L2

#### Phenytoin

Phenytoin exerts an anticonvulsant effect by blocking cell membrane sodium ion channels and is suitable for the treatment of generalized and focal epilepsy (25). Its molecular weight is 252, protein binding rate is about 89%, and distribution volume is 0.5–0.8L/kg (20). After taking the drug, time to peak level is about 4–12h (20). The absorption of phenytoin s unaffected by food and is mainly metabolized by CYP2C9 and CYP2C19. The half-life is about 22–40 h, the bioavailability is 95%, and about 5% of the drug is excreted through the urinary system in original form (20, 26, 27). Its permeability in breast milk is low. M/P ratio ranging from 0.18–0.45 and RID ranging from 0.6–7.7% (20). The evaluation of adverse reactions in infants exposed to phenytoin through breast milk found that in most cases, there were no serious adverse reactions (19). There a report that breastfeeding under treatment with phenytoin led to methemoglobinemia (28). But this is rare. The specific mechanism of phenytoin in breastmilk lead to methemoglobinemia in infants is still unclear. When some patients used phenytoin in combination with other ASMs (such as clomastatin and carbamazepine) during lactation, the baby had drowsiness, weight loss and other adverse reactions (29). In long-term follow-up, it was found that taking phenytoin during lactation would not cause cognitive damage to infants (30, 31).

#### Fosphenytoin

As a precursor of phenytoin, fosphenytoin was approved by the US Food and Drug Administration in 1996 for the treatment of acute symptomatic seizures and status epilepticus. It can be rapidly and completely converted to phenytoin after intramuscular or intravenous injection, with a bioavailability of 100%. The half-life is related to the mode of administration. It takes about 22–41 min for intramuscular injection and 8–15 min for intravenous administration (32–34). The molecular weight is 406, the protein binding rate is 99%, and the distribution volume is 0.06–0.15 L/kg. Time to peak level of fosphenytoin is 0.5 h (20). Because fosphenytoin plays a role *in vivo* by converting to phenytoin sodium, there is a lack of M/P ratio, RID and other data.

#### Carbamazepine

Carbamazepine is a voltage dependent sodium channel blocker used to control generalized tonic clonic seizures and focal seizures (35). Carbamazepine is catalyzed by cytochrome P450 (CYP) 3A4 and 3A5 and further metabolized to carbamazepine-10,11-epoxide (CBZE), which has a strong anticonvulsant effect. Subsequently, CBZE was hydrolyzed by microsomal epoxide hydrolase to the inactive metabolite carbamazepine-10,11-trans dihydrodiol. Only 1% of carbamazepine was excreted from urine in its original form, and its half-life is about 15 h (36, 37). Its molecular weight is 236, protein binding rate is 74%, and distribution volume is 0.8–1.8 L/kg (20). Time to peak level is 4–5 h after taking the drug (20). M/P ratio is about 0.69, and RID is 3.8–5.9% (20). The American Academy of Pediatrics believes that carbamazepine may be safe during breastfeeding (38). There is some anecdotal evidence that breastfeeding under treatment with carbamazepine led to side effects like jaundice, cholestasis and elevated liver transaminase (39). Another infant also had abnormal liver function (40). However, a small number of infants have problems such as poor sucking, vomiting and insufficient weight gain (41, 42).

#### Lamotrigine

Lamotrigine is one commonly used ASM, especially during pregnancy and breastfeeding (43). Its molecular weight is 256, the plasma protein binding force is about 55%, and the distribution volume is 0.9–1.3 L/kg (20). Its bioavailability is 98%. After about 3 h of oral administration, the blood drug concentration reaches the peak level, and then it is widely metabolized under the action of uridine 5 '- diphosphate glucuronosyltransferase family. Its bioavailability is 98%, and its half-life is about 29 h (44, 45). M/P ratio is 0.562, and RID is between 9.2–18.27% (20). It is reported that most mothers breastfeed when using lamotrigine, and their infants have no adverse reactions. However, a small number of patients have serious adverse reactions. For example, a case reported that breastfeeding under treatment with lamotrigine led to apnea (46). Some infants had abnormal liver function, inappetence, anemia, etc. (47, 48).

#### Levetiracetam

Levetiracetam is a synaptic vesicle protein 2A modulator that can be used to treat epilepsy in different seizure forms (49). Its molecular weight is 170, the oral bioavailability is up to 100%, and time of plasma drug concentration to peak level is 1h after oral administration (20). Levetiracetam is mainly hydrolyzed in blood and various tissues through B-type esterase and other secondary inactive metabolites. Its distribution volume is equal to 0.7 L/kg, plasma protein binding force is less than 10%, and its half-life is 6–8 h. When the renal failure, its half-life is prolonged (20, 50). M/P ratio equals 1, and RID is between 3.4 and 7.8% (20). In the previous reports, most infants exposed to levetiracetam through breastfeeding did not report any adverse reactions (51, 52). However, a small number of infants experienced somnolence, poor diet and other symptoms after contacting levetiracetam in breast milk. Fortunately, these adverse reactions returned to normal soon after breastfeeding was stopped (53, 54).

#### Gabapentin

Gabapentin is a GABA analog that acts on α-2 δ Binding sites, which combine with calcium channels (55). The molecular weight of the drug is 171, the oral bioavailability is only 50–60%, time to peak level is 1–3 h after oral administration, the distribution volume is 0.8 L/kg, and the protein binding force is <3% (20). Different from other ASMs, gabapentin is not metabolized in the body and almost completely excreted through the kidney. Its half-life is about 5–7 h, and its tolerance is well (20). M/P ratio is between 0.7–1.3, and RID is equal to 6.6% (20). Limited research shows that gabapentin in breastmilk does not cause serious adverse reactions to infants (51, 56, 57).

### Type L3

#### Oxcarbazepine

Oxcarbazepine is a derivative of carbamazepine. Its efficacy is similar to that of carbamazepine. It has a highly selective inhibitory effect on the movement of cerebral cortex. Its role is to block the sodium channel of cells, thus preventing the spread of epileptic focus discharge in the brain (58). Its molecular weight is 252. It is almost completely absorbed and utilized after oral administration. The time to peak level is 4.5 h. The distribution volume is 0.7 L/kg, and the protein binding force is 40% (20). Oxcarbazepine is rapidly reduced to clinically relevant metabolite 10,11-dihydro-10-hydroxy-cazepine through cytosolic enzymes in the liver (59). Its half-life is 2 h, but the half-life of metabolite 10,11-dihydro-10-hydroxy-cazepine is 9 h (20). M/P ratio is 0.5 (20). The RID is not mentioned in Hale (20) books, but other studies show that the RID of oxcarbazepine is between 1.5–1.7% (60). It is reported that most infants have no adverse drug reactions when they are exposed to oxcarbazepine through breastmilk. Even in the follow-up for several years, it is not found that oxcarbazepine in breastmilk will affect the growth and neurological development of infants (52, 61, 62).

#### Topiramate

Topiramate is a potent ASM with broad-spectrum activity that exerts anticonvulsant effects by modulating voltage-dependent sodium channels, enhancing gamma-aminobutyric acid (GABA) inhibition, blocking excitatory neurotransmission, and gating calcium channels (63). Its molecular weight is 339.4, the oral bioavailability is 80%, the time to peak level is 1.5–4h after oral administration, the distribution volume is 0.7 L/kg, and the protein binding force intervenes between 15–41% (20). *In vivo*, topiramate is metabolized through hydroxylation, hydrolysis and glucuronization. Topiramate is mainly excreted from urine in prototype form, with a half-life of about 21 h (63). M/P ratio is 0.86, and RID is between 24.68–55.65% (20). Although the RID of topiramate is more than 10%, limited studies have shown that topiramate in human milk does not cause serious adverse reactions in infants. There an evidence that breastfeeding under treatment with topiramate led to diarrhea (64).

#### Pregabalin

The molecular structure of pregabalin is very similar to GABA, which also acts on α- 2δ Binding sites and combines with calcium channels to play a role (65). Its molecular weight is 159, oral bioavailability is 90%, The time to peak level is 1.5 h after oral administration, the distribution volume is 0.5 L/kg, and it does not combine with plasma protein (20, 65). Pregabalin is hardly metabolized in the human body and excreted through the kidney. Its half-life is about 6 h (20, 65). M/P ratio is between 0.34–0.76, and RID is 7.18% (20). Limited data indicate very low levels of pregabalin in human milk. There is some anecdotal evidence that breastfeeding under treatment with pregabalin not led to side effects (66).

#### Lacosamide

Lacosamide is the third generation of new ASM, which can be used for the treatment of complex focal seizures, generalized seizures and status epilepticus (67). It slowly inactivates the selective enhanced voltage-gated sodium channe. In addition, it can also play an anticonvulsive effect by binding to the mediator protein 2 of the hydrolysis reaction (68). The molecular weight of the drug is 250, and the oral bioavailability is 100%. The time to peak level is 1–4 h with oral administration, the distribution volume is 0.6 L/kg, and the plasma protein binding capacity is about 15% (20). A portion of lacosamide is metabolized by CYP2C19, CYP2C9, and CYP3A4 with a half-life of approximately 13 h. Approximately 40% of lacosamide is cleared by the kidney as an unchanged active drug form (20, 69). Ruuskanen et al. showed that when the mother took lacosamide during lactation, the dose in the baby accounted for only 1.8% of the mother's dose (69). About the M/P ratio, only one study mentioned that it is about 0.83 (70). RID has not reported. However, most of the epileptic patients who took lacosamide during lactation did not find that the drug would cause cognitive changes or stunting of their children after breastfeeding (70, 71). There is some anecdotal evidence that breastfeeding under treatment with lacosamide and levetiracetam led to side effects like lethargy and poor diet (53).

### Type L4

#### Ethosuximide

Voltage gated calcium channels participate in the burst discharge of epileptic focus neurons and regulate the release of presynaptic membrane neurotransmitters, while ethosuximide inhibits the discharge of epileptic focus by blocking T-type calcium channels in thalamic cortical neurons for the treatment of absence seizures (35). The drug has a molecular weight of 141 and high oral availability. The time to peak level is 4 h after oral administration, and the distribution volume is 0.72 L/kg, without binding with plasma protein (20). It is mainly metabolized by CYP3A4. CYP2E and CYP2C/B are also involved in partial metabolism, ethosuximide has no induction effect on these enzymes, and the metabolites can be excreted with urine, with a half-life of about 30–60 h (20, 27). Its M/P ratio is 0.94 and RID is between 31.4–73.5% (20). There are few reports on adverse reactions caused by ethosuximide in breastmilk. It is mentioned in a paper that a mother breastfed with taking ethosuximide, which led to sedation, poor sucking and slow weight gain of infant (72).

#### Zonisamide

Zonisamide, a sodium channel blocker, is a second-generation ASM (35). The molecular weight of the drug is 212, and the time to peak level is 2–6 h after oral administration. The distribution volume is 1.45 L/kg, and the plasma protein binding force is about 40% (20). It is metabolized by CYP3A4 in the liver and excreted in the kidney with a half-life is about 63 h (20, 73). Studies had shown that zonisamide can be excreted into breastmilk in large quantities. The serum drug concentration in infants accounts for 44.2% of the maternal level, the M/P ratio is about 0.93, and RID is 28.88–44.1% (20, 43, 74). Despite the long half-life of zonisamide and the high penetration of breastmilk, no adverse events were reported in infants treated with zolesamide during breastfeeding.

#### Primidone

As a first-generation ASM, the mechanism of antiepileptic action of primidone is still unclear, but it is considered to be related to inhibition of voltage-gated sodium channels and extension of the opening time and frequency of chloride channels (75, 76). The molecular weight of the drug is 218, and the oral availability is 90%. The time to peak level is 1–2 h after oral administration, the distribution volume is 0.5–1 L/kg, and the plasma protein binding capacity is about 25% (20). It is metabolized by CYP2C9 and CYP2C19 isoenzymes in the liver and has a half-life of about 5–18 h (20, 77). M/P ratio is 0.72 and RID is 8.4–8.6% (20). Although the M/P ratio of primidone is less than 1 and RID is a safe range for intervention, the American pediatrics department believes that primidone may cause drowsiness and no weight gain in infants due to limited research data, so it should be used with caution during lactation (38).

#### Valproate

Valproate is a broad-spectrum anticonvulsant, which mainly increases the level of GABA in the brain or synapses to counter the excitatory effect of glutamate, so as to inhibit the abnormal discharge of brain neurons and achieve the purpose of seizure control. It can be used in the treatment of generalized epilepsy and focal epilepsy (78). The drug has a molecular weight of 144 and can be completely absorbed by oral administration. The time to peak level is 1–4 h after oral administration. The distribution volume is 0.1–0.4 L/kg, and the plasma protein binding force is about 94% (20). Valproate is metabolized primarily in the liver through glucuronidation, β-oxidative metabolism, and CYP-mediated oxidation, and excreted in the kidney with a half-life of approximately 14 h (20, 27). M/P ratio is 0.42 and RID is 0.99–5.6% (20). In most cases reported, valproate in breastmilk did not cause serious adverse effects in infants. In rare cases, some infants occurred side effects such as drowsiness, alopecia, petechiae, thrombocytopenia, anemia, and mild hematuria, and these symptoms gradually improve after drug withdrawal (79, 80).

#### Phenobarbital

Phenobarbital is a barbiturate with a long half-life that is commonly used as an anticonvulsant in the adult and neonatal period (20). The molecular weight of the drug is 232, about 80% of phenobarbital can be absorbed orally, and the time to peak level is 0.4–0.6 h after oral administration, the distribution volume is 0.5–0.6L/kg, and the plasma protein binding capacity is about 51% (20). It is mainly metabolized by the hepatocytes CYP2C9, CYP2C19, and CYP2E1, and the bound metabolites are cleared by the kidney. About 25% of phenobarbital is excreted in the urine in its original form with a half-life of about 53–140 h (20, 81). M/P ratio is 0.4–0.6 and RID is 24% (20). It has been reported that phenobarbital in breastmilk can cause drowsiness in infants (82). Because RID is higher than 10% and the half-life is long, the American Academy of Pediatrics believes that phenobarbital should be used with caution during lactation (38).

#### Clobazam and clonazepam

Although some scholars previously believed that clobazam and clonazepam belonged to L3 (20), a recent report suggested that they should belong to L4 (83). Clobazam and clonazepam are benzodiazepines, mainly used as adjunctive therapy for epilepsy (84). The molecular weight of clobazam is about 301, the oral bioavailability is 87%, the time to peak level is 0.5–4 h after oral administration, the distribution volume is 1.43 L/kg, and the plasma protein binding force is between 80–90% (20). It is mainly metabolized by CYP 3A4 and 2C19, with a half-life is about 36–42 h (15, 20). M/P ratio is 0.13–0.36, and RID is 4.38–5.33% (20). The molecular weight of clonazepam is about 316, which can be quickly absorbed after oral administration. The time to peak level is 1–4 h, the distribution volume is between 1.5–4.4 L/kg, and the plasma protein binding force is between 50–86% (20). It is mainly metabolized by CYP 3A4, with a half-life is about 18–50 h (15, 20). M/P ratio is 0.33, and RID is 2.8% (20). Benzodiazepines in breast milk may cause sedation to infants, so it is necessary to monitor whether infants have drowsiness during use (85).

### Other ASMs

For some of the ASMs, due to the lack of clinical research data, there is no clear classification.

#### Rufinamide

Rufinamide inhibits the hyperexcitability of neurons by prolonging the inactivation phase of voltage gated sodium channel (86, 87). We have described the pharmacokinetics of the drug in Table 1. There is a lack of information on the use of rufinamide during breastfeeding (88).

#### Stiripentol

Stiripentol is used for the treatment of Dravet syndrome. It is a positive allosteric regulator of GABA-A receptor, which plays an anticonvulsant effect by increasing receptor sensitivity (89, 90). It is rapidly absorbed after oral administration, and the plasma protein binding rate is 99%. It is mainly metabolized by oxidation, hydroxylation, O-methylation and glucuronidation, and a small amount is excreted by the kidney (91). It is a potent inhibitor of CYP3A4, 1A2 and 2C19. When used with other ASMs, we should pay attention to monitoring the serum concentration of the drug (74). At present, there is no data on the serum drug concentration of stiripentol in breastmilk and infants and no literature data on the use of stiripentol in lactation (88).

#### Perampanel

Perampanel plays an anticonvulsive role by selectively blocking α-amino-3-hydroxy-5-methyl-4-isoxazolpropionate receptors, limiting glutamate binding to receptors and reducing excitatory neurotransmitter transmission, thereby limiting the ability of epileptogenic foci to discharge and spread (35). As a new generation of ASM, it has the advantages of long half-life and can penetrate the blood brain barrier (92, 93). Perampanel is mainly metabolized through cytochrome P450 3A4 (CYP3A4) and CYP3A5, and CYP3A4. Unlike other ASMs, its metabolites are mainly excreted with feces, and a small part is excreted with urine (92). There are few studies on the concentration of perampanel in breastmilk. A limited case report showed that mother took brivaracetam, lacosamide and perampanel at the same time during the whole pregnancy. It found that the serum concentration of perampanel increased significantly during the whole pregnancy and after delivery. However, in the follow-up of infants, the serum concentration at 11 weeks after birth was decrease, and the average M/P ratio was 0.13 (70).

#### Brivaracetam

Similar to levetiracetam, brivaracetam is also a regulator of synaptic vesicular protein 2A, but its affinity is 10–30 times that of levetiracetam. It is used to control focal seizures in clinic (94). After oral administration, it is rapidly absorbed through the gastrointestinal tract and metabolite is excreted through the kidney (95, 96). In liver dysfunction, the metabolic clearance rate of brivaracetam is reduced. Some studies showed that the maximum daily dose of brivaracetam in patients with liver function damage may need to be reduced by one third (97). Therefore, in patients with liver dysfunction, the metabolic clearance rate of brivaracetam is reduced, and we should be paid to reducing the drug dose. There are few reports on breastfeeding of brivaracetam. Eylert Brodtkorb et al. followed up a patient who used brivaracetam during pregnancy (75 mg bid) and found that the serum concentration of brivaracetam in infants during exclusive breastfeeding did not change at birth, the 5th day and the 5th week, accounting for 18–20% of the mother's body, and M/P ratio was 0.71 (70).

#### Cannabidiol

Cannabidiol can be used in Dravet syndrome and Lennox-Gastaut syndrome (97). At present, the mechanism of cannabinol's anticonvulsant effect is not clear, but many studies suggest that it is related to the antagonistic G protein coupled receptor 55, the transient receptor potential desensitization of vanillin type 1 channel and the inhibition of adenosine reuptake (98). After oral administration, it is extensively metabolized in the liver and intestine mainly through CYP2C19 and CYP3A4, UGT1A7, UGT1A9, and UGT2B7. Metabolites are mainly excreted through the gastrointestinal tract, and a small part is excreted with urine (99). At present, there is no data on the use of cannabinol by epileptic patients during lactation, but in the breastmilk of other patients who have used cannabis, it is found that there is still a metabolite of cannabis in the breast milk of mothers who have used cannabis for 6 days (100).

#### Eslicarbazepine acetate

Eslicarbazepine acetate inhibits the discharge of seizure focus by inhibiting the activity of voltage gated sodium channel (101, 102). Eslicarbazepine acetate is well absorbed in the gastrointestinal tract. After oral administration, it is metabolized by the liver and then converted into eslicarbazepine, which is excreted by the kidney. Its half-life is long. It only needs to be administered once a day. After repeated administration, it reaches the stable level 4–5 days later (103, 104). Some studies have shown that the drug can be measured in animal breastmilk, but no study has been made to determine whether it will be metabolized into human breastmilk (105).

Except for special instructions, other citations are described in the paper.

## Conclusion

For most women with epilepsy, taking ASMs during their reproductive years is unavoidable. More importantly, mothers with epilepsy also need to face an important issue on whether they can breastfeed after delivery. These are difficult choices for patients to make. In the past, using ASMs during lactation was considered contraindicated for breastfeeding because of the possibility of indirect exposure of infants to ASMs through breastmilk (106). However, in recent years, after balancing the benefits of breastfeeding, mothers receiving ASM treatment have been actively encouraged to start breastfeeding immediately after delivery (60, 83). It is complex to assess the breastfeeding risk of any drug, and neither pharmacokinetics, M/P ratio, nor RID alone are good predictors of breastfeeding risk (60).

Carbamazepine in breastmilk may cause vomiting, sucking difficulties, insufficient weight gain, elevated transaminase, jaundice and cholestasis in infants. Lamotrigine may cause lethargy, food refusal, apnea, elevated liver enzymes and jaundice in infants. Levetiracetam may cause infants to fall asleep and refuse to eat. Phenytoin may cause drowsiness, anorexia and methemoglobinemia. Benzodiazepines may cause apnea, decreased muscle tone and sedation in infants. Topiramate may cause diarrhea. Oxcarbazepine may be associated with increased excitability, irritability, limb tremor and increased muscle tone. Lacosamide may be manifested as lethargy, poor diet, etc. Valproic may cause ecchymosis, thrombocytopenia, anemia and hematuria in infants. Ethosuximide and primidone may cause drowsiness, refusal to eat and weight loss. No adverse reactions were reported for gabapentin, phenytoin, pregabalin and zonisamide. According to pharmacokinetics, their safety assessment classification in breastfeeding is different (Table 1).

In conclusion, taking ASMs is not a contraindication to breastfeeding. It is important to observe a baby's daily condition.

However, this paper also has some limitations. For some of the ASMs, due to the lack of relevant clinical researches, this paper cannot give advice on the use of related drugs.

## Author contributions

RY, ZT, HZ, and JY designed and wrote the manuscript. JT checked words and grammar. ZX and CY helped with proofreading and revision. All authors contributed to the article and approved the final version.

## Funding

This work was supported by the Collaborative Innovation Center of the Chinese Ministry of Education (2020-39) and the Guizhou Provincial Hundred Level Innovative Talents Funds (No: GCC-2022-038-1).

## Conflict of interest

The authors declare that the research was conducted in the absence of any commercial or financial relationships that could be construed as a potential conflict of interest.

## Publisher's note

All claims expressed in this article are solely those of the authors and do not necessarily represent those of their affiliated organizations, or those of the publisher, the editors and the reviewers. Any product that may be evaluated in this article, or claim that may be made by its manufacturer, is not guaranteed or endorsed by the publisher.
